# Supported Nanostructured Mo_x_C Materials for the Catalytic Reduction of CO_2_ through the Reverse Water Gas Shift Reaction

**DOI:** 10.3390/nano12183165

**Published:** 2022-09-13

**Authors:** Arturo Pajares, Xianyun Liu, Joan R. Busacker, Pilar Ramírez de la Piscina, Narcís Homs

**Affiliations:** 1 Departament de Química Inorgànica i Orgànica, Secció de Química Inorgànica & Institut de Nanociència i Nanotecnologia (IN2UB), Universitat de Barcelona, Martí i Franquès 1, 08028 Barcelona, Spain; 2 Catalonia Institute for Energy Research (IREC), Jardins de les Dones de Negre 1, 08930 Barcelona, Spain

**Keywords:** CO_2_ catalytic reduction, syngas, RWGS, supported molybdenum carbide, Mo_x_C-based catalysts

## Abstract

Mo_x_C-based catalysts supported on γ-Al_2_O_3_, SiO_2_ and TiO_2_ were prepared, characterized and studied in the reverse water gas shift (RWGS) at 548–673 K and atmospheric pressure, using CO_2_:H_2_ = 1:1 and CO_2_:H_2_ = 1:3 mol/mol reactant mixtures. The support used determined the crystalline Mo_x_C phases obtained and the behavior of the supported nanostructured Mo_x_C catalysts in the RWGS. All catalysts were active in the RWGS reaction under the experimental conditions used; CO productivity per mol of Mo was always higher than that of unsupported Mo_2_C prepared using a similar method in the absence of support. The CO selectivity at 673 K was above 94% for all the supported catalysts, and near 99% for the SiO_2_-supported. The Mo_x_C/SiO_2_ catalyst, which contains a mixture of hexagonal Mo_2_C and cubic MoC phases, exhibited the best performance for CO production.

## 1. Introduction

In addition to capture and storage of CO_2_, nowadays there is a clear interest in its use as an out-stream chemical feedstock in order to actively contribute to the reduction of CO_2_ emissions; CO_2_ can be considered a cheap carbon C1 source for upgrading rather than a waste with consequences in global warming [1,2,3,4]. However, the direct transformation of CO_2_ to useful products is difficult. The high chemical stability of CO_2_ difficult its catalytic transformation, the developing of new materials capable of efficiently bind and activate this molecule is nowadays an active research area. An interesting CO_2_ utilization approach is its reduction to CO, employing H_2_ as a reducing agent via the reverse water gas shift (RWGS) reaction [5,6,7,8]:

CO_2_ + H_2_ → CO + H_2_O (1)

The reduction of CO_2_ to CO with renewable H_2_ can be regarded as a simple and easy path for CO_2_ recycling, which would allow its reuse at a large scale. After the RWGS step and H_2_O separation, a CO_2_/CO/H_2_ out-stream mixture can be produced. This out-stream can be used as syngas input for other well-established chemical processes, such as Fischer-Tropsch (FT) or methanol synthesis [9,10,11,12,13,14,15].

The RWGS reaction can be carried out using noble metal-based catalysts [5,10,16]. Due to the similar properties of transition metal carbides (TMCs) and Pt-based catalysts, the formers have been proposed as catalysts for different processes in which Pt-based catalysts are active [17,18]. One of these processes is the CO_2_ reduction to CO, which has been analyzed over different TMCs using theoretical and experimental approaches [19,20,21,22,23,24,25]. 

The preparation of TMCs is usually carried out using carburization methods. These methods apply high temperature and/or pressure conditions in the presence of a reducing atmosphere, usually mixtures of H_2_ and carbon-containing gases (CO, CH_4_, C_2_H_4_) [25,26,27,28]. Due to the increased interest in TMC-based catalysts, in recent years, greener preparation methods have been explored [21,22,29,30,31]. In an earlier investigation, we studied the preparation of bulk Mo_x_C catalysts using different molybdenum and carbon precursors and following sol-gel based routes; the bulk Mo_x_C catalysts generated, contained different crystalline phases, which influenced their catalytic behavior in the RWGS reaction [31]. 

The deposition onto a support of the appropriate TMC active phase can be an interesting approach to improve the catalytic behavior of bulk TMCs materials, which usually show low surface area values. Supported Mo_x_C phases have been used as catalysts in different processes such as CH_4_ dry reforming [32], hydrazine decomposition [33], thiophene hydrodesulfurization [34], propene and tetralin hydrogenation [35] and Fischer-Tropsch synthesis [36]. However, supported Mo_x_C catalysts have not been much studied in the RWGS reaction [37,38,39]. Porosoff et al. have reported the promoter effect of K in Al_2_O_3_- supported Mo_2_C-based catalysts containing MoO_2_ and/or metallic Mo, which were prepared by carburization with CH_4_/H_2_ at 873 K [38]. Sub-nanosized molybdenum carbide clusters highly dispersed onto N-doped carbon/Al_2_O_3_, prepared by carbonization of MoO_3_ with glucose, were more performant in the RWGS than bulk β-Mo_2_C [39]. Recently, the preparation of SiO_2_- and SBA-15-supported Mo_2_C-based catalysts (20% wt Mo), using different routes of Mo incorporation to the support and a final carburization process with CH_4_/H_2_, has been studied [40]. The preparation method and the support influenced the composition of Mo_x_C_y_ crystalline phases developed and therefore the catalytic performance of the material in the RWGS [40]. The preparation of Mo_x_C-based catalysts supported onto γ-Al_2_O_3_, SiO_2_ and MFI-type zeolites by incipient wetness impregnation of ammonium molybdate and carburization with CH_4_/H_2,_ have led to catalysts with different Mo-containing species such as Mo_2_C, MoO_3_ and Mo^0^; the phases developed and the catalytic performance in the RWGS of the materials depended also on the support characteristics [41].

Here, Mo_x_C phases were generated onto γ-Al_2_O_3_, SiO_2_ and TiO_2_ by a thermal treatment of the solid obtained from the interaction between a MoCl_5_/urea solution and the corresponding oxide. The crystalline Mo_x_C phases obtained depended on the support used in the preparation and determined the catalytic behavior of materials in the RWGS.

## 2. Experimental

### 2.1. Preparation of Catalysts

Commercial γ-Al_2_O_3_ (Alfa Aesar, Haverhill, MA, US, 226 m^2^ g^−1^), SiO_2_ (Degussa, Frankfurt, Germany, 200 m^2^ g^−1^) and TiO_2_ (Tecnan, Navarra, Spain, 117 m^2^ g^−1^, anatase/rutile, 78/22% wt) were employed as supports. Urea (Alfa Aesar, Haverhill, MA, US, 99%), which was used as carbon source, was added to a solution of MoCl_5_ (Alfa Aesar, Haverhill, MA, US, 99.6%) in ethanol with a urea/MoCl_5_ = 7 molar ratio [21,29,31]. The viscous solution was contacted with the respective powdered support. The resulting solid was dried at 333 K, and then treated under Ar flow up to 1073 K for 3 h. The samples were cooled down to room temperature under Ar and then exposed to air without passivation. Mo_x_C/Al_2_O_3_, Mo_x_C/TiO_2_ and Mo_x_C/SiO_2_ catalysts with about 26% wt of Mo were prepared by using the proper amount of molybdenum and carbon precursors. A reference catalyst (unsupported), containing only bulk hexagonal Mo_2_C was prepared following a similar method but in the absence of support [21]. For characterization purposes, the commercial supports were also separately treated up to 1073 K (3 h) under Ar.

### 2.2. Characterization of Catalysts

The Mo content of samples was determined by inductively coupled plasma mass spectrometry using a Perkin Elmer Optima 3200RL apparatus (Santa Clara, CA, US). The N_2_ adsorption-desorption isotherms were recorded at 77 K using a Micromeritics Tristar II 3020 equipment. Prior to the measurements, the samples were outgassed at 523 K for 5 h. The specific surface area (S_BET_) was calculated by multi-point BET analysis of N_2_ adsorption isotherms. The X-ray powder diffraction (XRD) analysis was performed using a PANalytical X’Pert PRO MPD Alpha1 powder diffractometer (Malvern, UK) equipped with a CuKα_1_ radiation. The XRD profiles were collected in the 2θ range of 4°–100° with a step size of 0.017° and counting 50 s at each step. Transmission electron microscopy (TEM-HRTEM) images and energy dispersive X-ray analysis (EDX) were collected employing a JEOL J2010F microscope (Tokyo, Japan) operated at an accelerating voltage to 200 kV. The Raman spectra of the samples were collected using a Jobin-Yvon LabRam HR 800, fitted to an optical Olympus BXFM microscope (Kyoto, Japan) with a 532 nm laser and a CCD detector. X-ray photoelectron spectroscopy (XPS) analysis was performed using a Perkin Elmer PHI-5500 Multitechnique System (Physical Electronics, Chanhassen, MN, US) with an Al X-ray source (hυ = 1486.6 eV and 350 W). Samples were kept in an ultra-high vacuum chamber during data acquisition (5·10^−9^–2·10^−8^ Torr). Before XPS measurements, the C 1s BE of adventitious carbon was determined in the same equipment and conditions using Au as reference. The BE values were referred to the mentioned C 1s BE at 284.8 eV.

### 2.3. RWGS Catalytic Tests

The RWGS reaction tests were carried out in a Microactivity-Reference unit (PID Eng&Tech) using a tubular fixed-bed reactor under atmospheric pressure. Approximately, 150 mg of catalyst were diluted with inactive SiC up to 1 mL of catalytic bed. The RWGS was studied at 0.1 MPa, between 548 K and 673 K, by following the temperature sequence: 598 K (3 h)→573 K (3 h)→548 K (10 h)→598 K (3 h)→623 K (3 h)→648 K (3 h)→673 K (3 h)→648 K (5 h). The first part of the catalytic test: 598 K (3 h)→573 K (3 h)→548 K (10 h) was carried out in order to condition the catalyst under RWGS. The gas hourly space velocity (GHSV) was 3000 h^−1^. The effluent was analysed on-line with a gas chromatograph Varian 450-GC equipped with a methanizer and TCD and FID detectors. CO_2_ conversion and product distribution at each temperature were determined by the average of at least three measures.

## 3. Results and Discussion

As stated above, Al_2_O_3_-, SiO_2_- and TiO_2_-supported Mo_x_C catalysts with about 26% wt Mo were prepared, characterized and tested in the RWGS reaction. Table 1 shows the Mo content and the S_BET_ of fresh catalysts. For comparison, S_BET_ values of the supports treated at 1073 K under Ar, which are the conditions used in the preparation of catalysts, are also included. In all cases, the S_BET_ of the supports after the thermal treatment at 1073 K was lower than that of the corresponding commercial pristine material; the diminution was about 10% for Al_2_O_3_ and SiO_2,_ meanwhile for TiO_2_ the S_BET_ decreased from 117 m^2^g^−1^ to 13 m^2^g^−1^. For TiO_2_, a phase change occurred during the thermal treatment; the rutile weight percentage increased from 22% (pristine material) until 95% after the treatment at 1073 K, as determined from XRD analysis [42]. On the other hand, except for the Mo_x_C/TiO_2_, the S_BET_ of supported catalysts was lower than that of the corresponding support treated at 1073 K; the formation of Mo_x_C could prevent in some extension the surface area decrease of the TiO_2_ support, which could be related with a different extent of the rutile formation from anatase.

**Figure 1:**
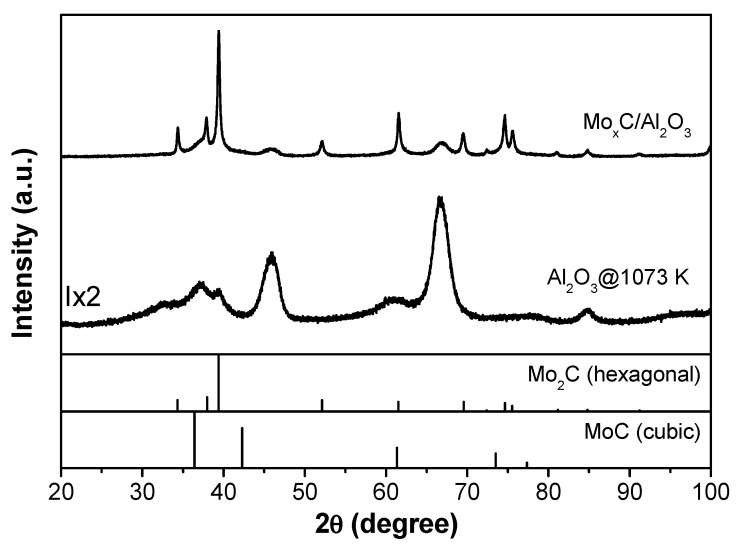
XRD patterns of Mo_x_C/Al_2_O_3_ catalyst and the Al_2_O_3_ support after thermal treatment at 1073 K.

**Figure 2:**
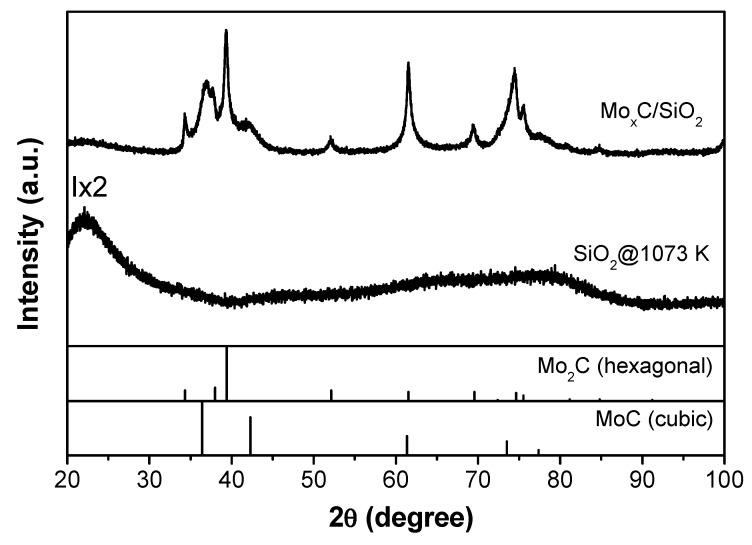
XRD patterns of Mo_x_C/SiO_2_ catalyst and the SiO_2_ support after thermal treatment at 1073 K.

**Figure 3:**
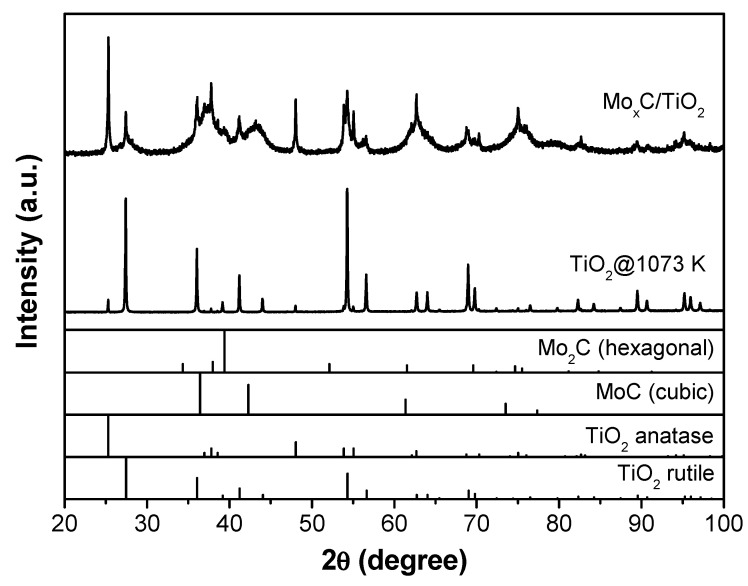
XRD patterns of Mo_x_C/TiO_2_ catalyst and the TiO_2_ support after thermal treatment at 1073 K.

The supported catalysts were analyzed by XRD, and the corresponding XRD patterns are shown in Figure 1, Figure 2 and Figure 3; XRD patterns of the respective supports treated at 1073 K under Ar are also displayed for comparison. From the XRD pattern of Mo_x_C/Al_2_O_3_ (Figure 1), characteristic diffraction peaks of γ-Al_2_O_3_ are observed, and the main presence of hexagonal Mo_2_C (JCPDS 00-035-0787) can be deduced; a crystallite size of 28 nm was calculated. The XRD analysis of Mo_x_C/SiO_2_ (Figure 2) indicates the presence of hexagonal Mo_2_C; however, the observation of diffraction peaks with maxima at 2θ = 36.9° and 2θ = 42.1° are attributed to the presence of cubic MoC (JCPDS 03-065-0280). From the intensity of diffraction peaks of both phases and that in reference files, a semiquantitative analysis was performed [43]; the presence of 65% cubic MoC and 35% hexagonal Mo_2_C is determined in the Mo_x_C/SiO_2_ catalyst. Figure 3 shows the corresponding XRD profile of TiO_2_-supported catalyst. Characteristic diffraction peaks of both anatase and rutile TiO_2_ phases are clearly observed. The rutile weight percentage with respect to TiO_2_ phases calculated from XRD pattern is 51% [42]. As commented above, the formation of Mo_x_C could prevent the anatase transformation, having the Mo_x_C/TiO_2_ catalyst a higher amount of anatase and a higher surface area than the support treated at 1073 K (Table 1). From the XRD pattern of Mo_x_C/TiO_2_, the main presence of cubic MoC with poor crystallinity can be proposed, even if the presence of hexagonal Mo_2_C could not be ruled out (Figure 3).

The catalysts were also characterized by Raman spectroscopy, TEM-HRTEM, STEM-EDX and XPS. Raman spectroscopy was used in order to determine the presence of molybdenum oxide species and/or carbonaceous residues (Figure S1). The very low intensity bands in the zone 815–990 cm^−1^ points to the presence of residual MoO_3_ [44,45,46], which could be formed by surface oxidation when the samples were exposed to air. For Mo_x_C/TiO_2_, Raman bands at 260, 429 and 610 cm^−1^, assigned to rutile, and at 150 cm^−1^ assigned to anatase, are clearly visible [47,48,49]. In all cases, the intensity of the bands in the 1200–1700 cm^−1^ region characteristic of carbonaceous species (D and G bands), is negligible (Figure S1).

**Figure 4:**
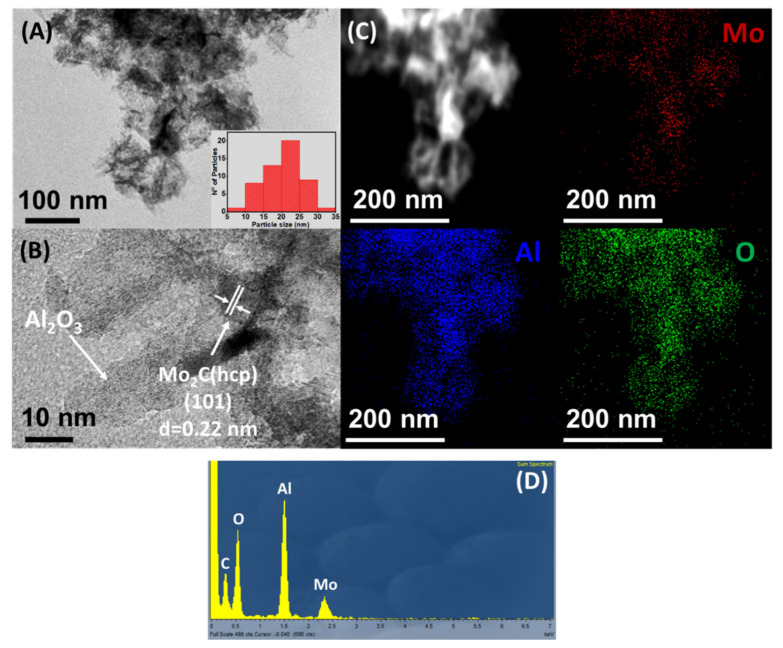
(**A**,**B**) TEM−HRTEM micrographs of Mo_x_C/Al_2_O_3_ catalyst; (**C**) STEM−EDX mapping; (**D**) EDX spectrum.

**Figure 5:**
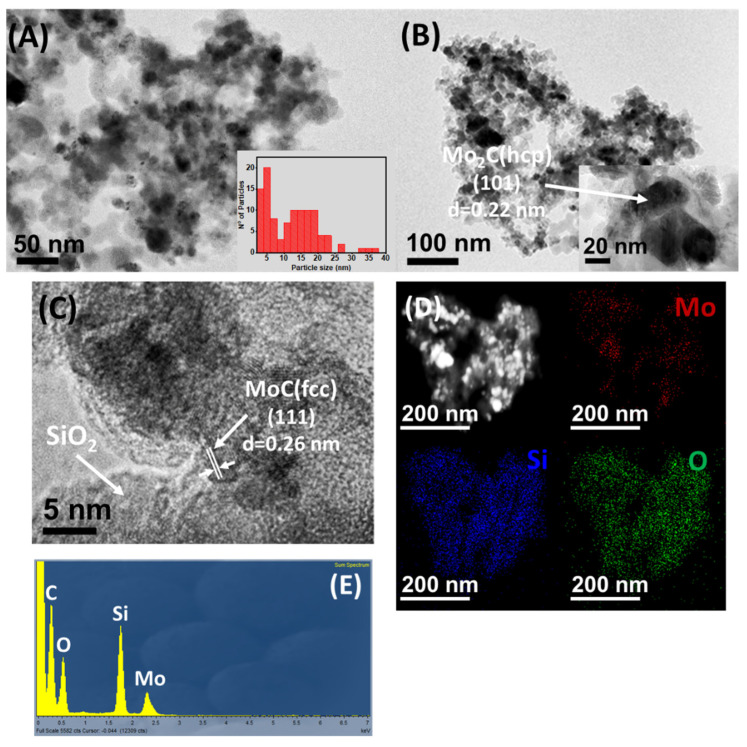
(**A**–**C**) TEM−HRTEM micrographs of Mo_x_C/SiO_2_ catalyst; (**D**) STEM−EDX mapping; (**E**) EDX spectrum.

**Figure 6:**
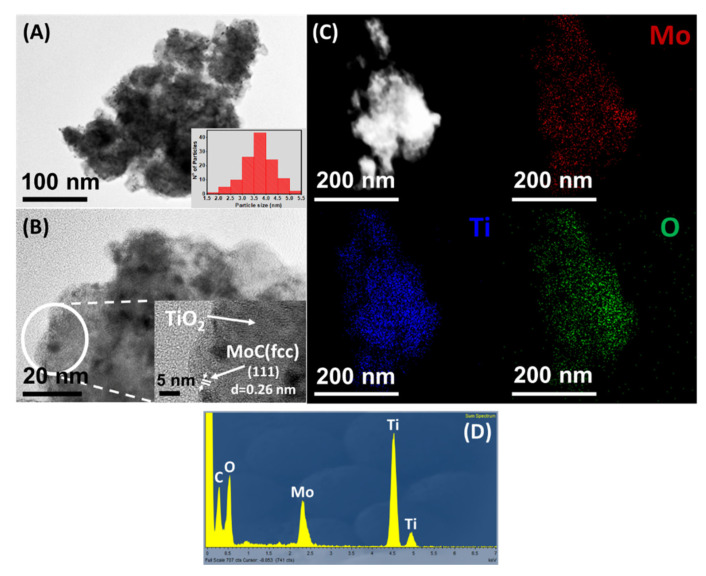
(**A**,**B**) TEM−HRTEM micrographs of Mo_x_C/TiO_2_ catalyst; (**C**) STEM−EDX mapping; (**D**) EDX spectrum.

TEM-HRTEM and STEM-EDX analysis of Mo_x_C/Al_2_O_3_, Mo_x_C/SiO_2_ and Mo_x_C/TiO_2_ are shown in Figure 4, Figure 5 and Figure 6, respectively. For Mo_x_C/Al_2_O_3_ (Figure 4), the presence of hexagonal Mo_2_C with a mean particle size of 21 nm was determined in agreement with XRD results. TEM-HRTEM analysis of Mo_x_C/SiO_2_ (Figure 5) allowed to confirm the presence of hexagonal Mo_2_C and cubic MoC particles with bimodal distribution and mean particle sizes of 18 nm and 5 nm, respectively (Figure 5A–C). For Mo_x_C/TiO_2_ (Figure 6), only the presence of the cubic MoC phase with a mean particle size of 4 nm could be determined. The supported Mo_x_C materials studied in this work follow the recently predicted general trend of size-dependent phase diagrams for bulk Mo and W carbides: fcc phases are generally found at small particle size and hcp phases are prevalent at large particle size [50]. 

In all cases, STEM-EDX results (see Figure 4C, Figure 5D, and Figure 6C) indicate a homogeneous distribution of Mo on the corresponding support. Figure 4D, Figure 5E and Figure 6D, show the corresponding EDX spectra; N- and Cl-containing species were not detected.

**Figure 7:**
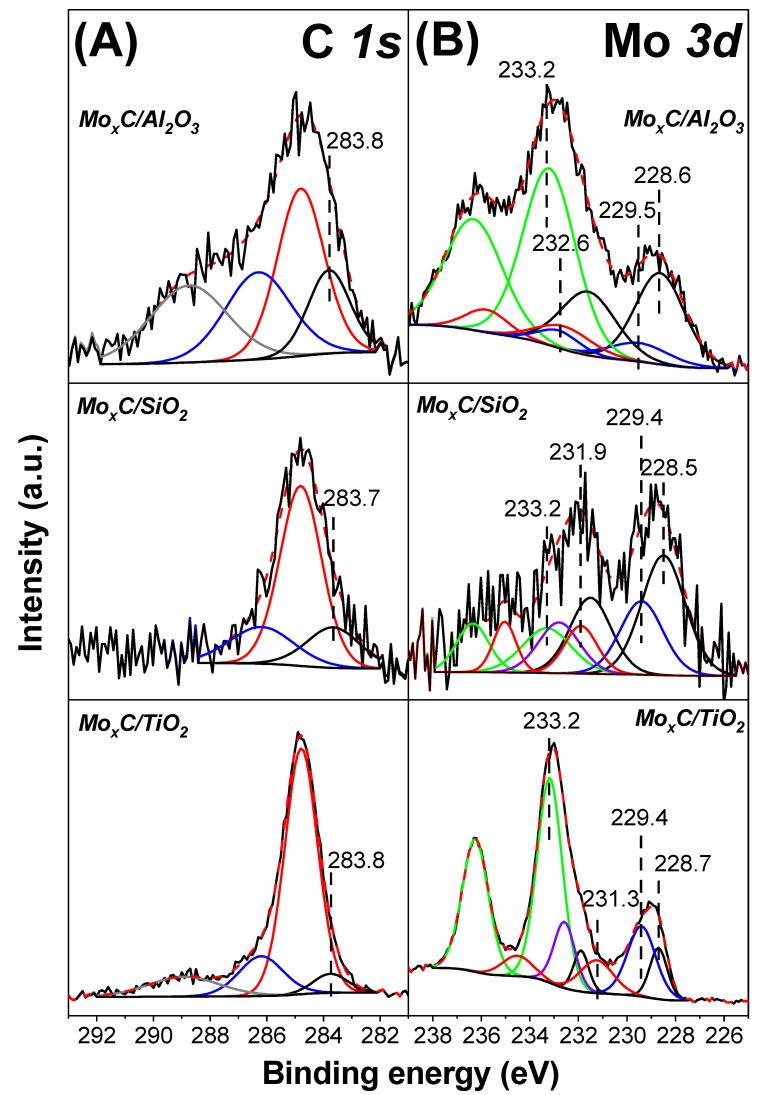
XP spectra of Mo_x_C/support catalysts: (**A**) C 1s level; (**B**) Mo 3d level.

As stated above, the catalysts were also analyzed by XPS. Al 2p, Si 2p and Ti 2p_3/2_ BE at 74.8, 104,0 and 459,3 eV, characteristic of Al_2_O_3_, SiO_2_, and TiO_2_, were found for Mo_x_C/Al_2_O_3_, Mo_x_C/SiO_2_ and Mo_x_C/TiO_2_, respectively (Figure S2). Figure 7 shows the C 1s and Mo 3d XP spectra. The C 1s core level spectra (Figure 7A) show a maximum at 284.8 eV associated to the adventitious carbon, the component at 283.7–283.8 eV is associated to surface molybdenum carbide species [21,31,51,52,53,54]. Components extended above 284.8 eV are related to different oxygen containing species [52,53,54,55,56]. The Mo 3d spectra are complex (Figure 7B); however, they can be deconvoluted into four doublets (Mo 3d_5/2_ and Mo 3d_3/2_). According to literature, the Mo 3d_5/2_/Mo 3d_3/2_ intensity ratio was fixed to be 1.5, and the Mo 3d_5/2_-Mo 3d_3/2_ BE splitting was set at 3.1 eV [57,58,59]. The 3d_5/2_ peaks at the lowest BE region, 228.5–228.7 eV, are attributed to Mo^2+^ and Mo^3+^ in Mo_2_C and/or oxycarbide species [19,21,31,51]. The Mo 3d_5/2_ components at 229.4–229.5, 231.3–232.6 and 233.2 eV, can be assigned to Mo^4+^, Mo^5+^ and Mo^6+^ surface species, respectively [19,58,59,60,61], which could be related to the presence of MoC, oxycarbide and/or oxide species. Table 2 shows the contribution of Mo^2+^/Mo^3+^ and Mo^4+^ species to the total surface Mo^n+^ species; the Mo_x_C/SiO_2_ catalyst having both Mo_2_C and MoC shows the highest values.

**Figure 8:**
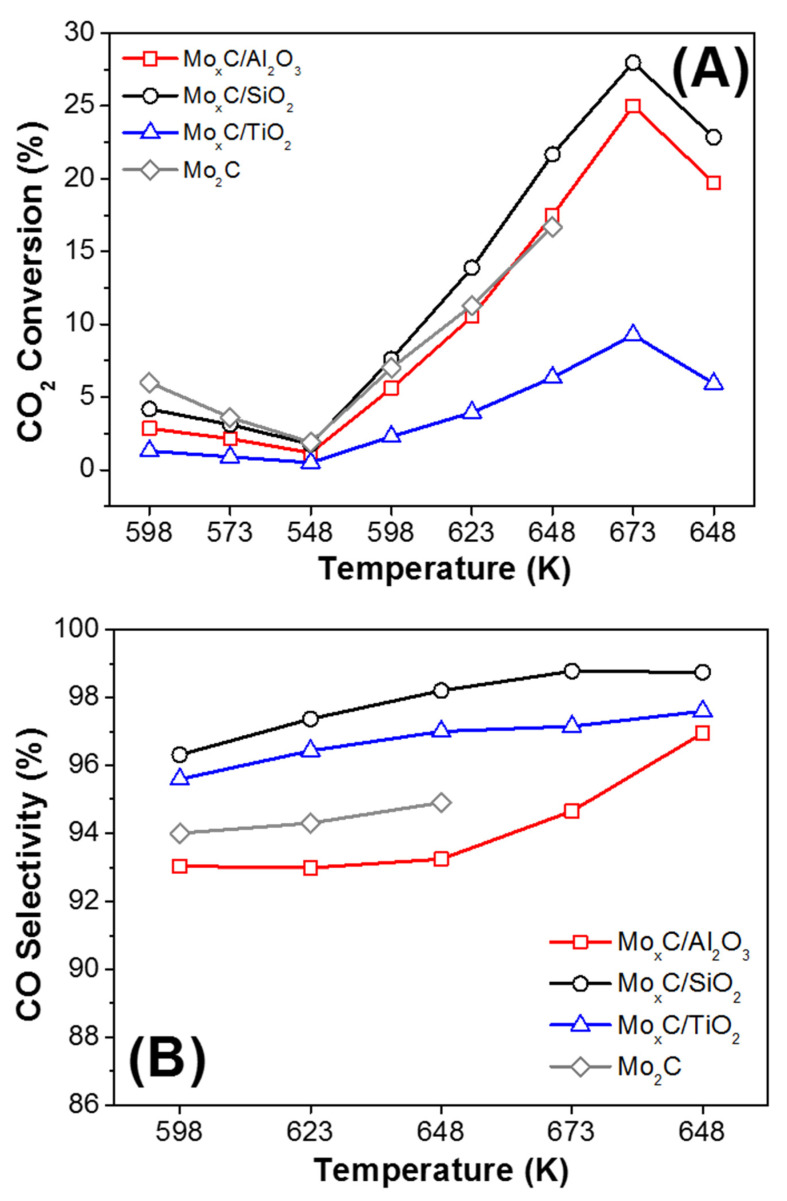
Catalytic behavior of Mo_x_C/support and unsupported reference Mo_2_C catalysts in the RWGS reaction as a function of reaction temperature; (**A**) CO_2_ conversion, (**B**) CO selectivity. Reaction conditions: m_cat_ = 150 mg, CO_2_/H_2_/N_2_ = 1/3/1, GHSV = 3000 h^−1^, P = 0.1 MPa.

All catalysts were tested in the RWGS using CO_2_:H_2_ = 1/3 and CO_2_/H_2_ = 1/1 ratios. Catalytic data of unsupported Mo_2_C, prepared using a similar method to that used in this work but in the absence of support, are also included for comparison [21]. As stated in the experimental section, the first part of the catalytic test: 598 K (3 h)→573 K (3 h)→548 K (10 h) was carried out in order to condition the catalyst under RWGS. Next, when the temperature was increased to 598 K, the CO_2_ conversion was in all cases higher than that obtained at 598 K in the conditioning step (Figure 8A and Figure 10A). This behavior could be related with the removal of initially adsorbed surface species. After this first step and regardless the catalyst and the conditions, CO_2_ conversion increases with the rising of reaction temperature from 598 K to 673 K (Figure 8A and Figure 10A). 

**Figure 9:**
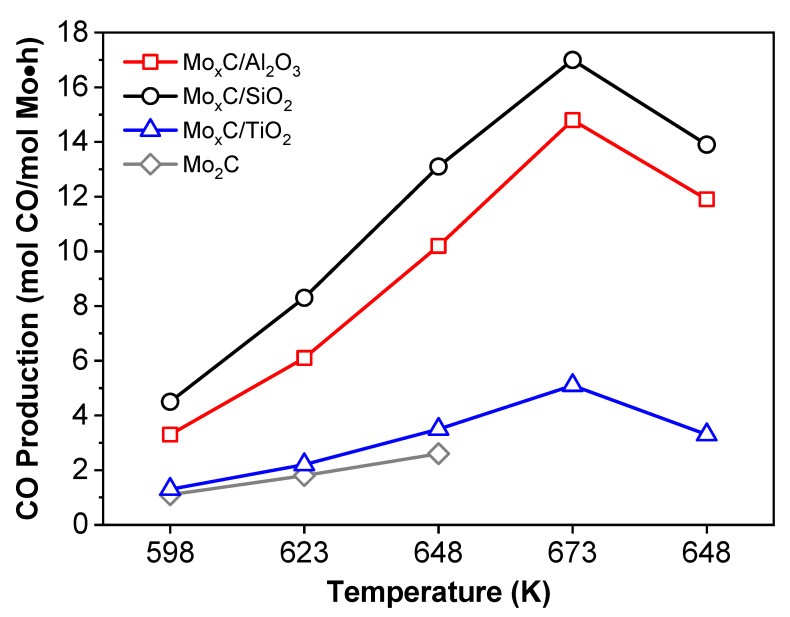
CO production per mol of Mo as a function of reaction temperature in RWGS over Mo_x_C/support and unsupported reference Mo_2_C catalysts. Reaction conditions: m_cat_ = 150 mg, CO_2_/H_2_/N_2_ = 1/3/1, GHSV = 3000 h^−1^, P = 0.1 MPa.

Figure 8 and Figure 9 show the RWGS behavior of catalysts when CO_2_:H_2_ = 1/3 is used. Mo_x_C/SiO_2_ presented the highest value of CO_2_ conversion (27.5%) at 673 K (Figure 8A); the corresponding equilibrium CO_2_ conversion for RWGS at the experimental conditions used is about 37% (at 673 K). Mo_x_C/Al_2_O_3_ showed a catalytic activity close to that of the unsupported Mo_2_C catalyst. Meanwhile, Mo_x_C/TiO_2_ showed lower values of CO_2_ conversion than those of unsupported Mo_2_C [21]. These results contrast with those usually reported for supported metallic catalysts [62,63]. The activity of SiO_2_- and Al_2_O_3_-supported metals in the RWGS is usually lower than that found when reducible supports such as TiO_2_ or CeO_2_ are used, which can generate oxygen vacancies that strengths the CO_2_ adsorption and then the activity in the RWGS [63]. In this work, besides the difference in the surface-area of catalysts, the composition and characteristics of generated Mo_x_C nanoparticles change as a function of the support. 

A key process in the RWGS is the cleavage of C-O bond with CO + O formation. In this context molybdenum oxycarbide has been proposed as an intermediate in the RWGS over Mo_2_C that likely enhances the RWGS rate [25]. We have demonstrated that over a polycrystalline α-Mo_2_C catalyst, prepared with the method used in the present work, the enhanced CO_2_ dissociation toward CO + O results from specific surface facets [21]. Next, the easy release of CO and the continuous O removal by H_2_ to form H_2_O, results in high RWGS activity. The existence of both, hcp Mo_2_C and fcc MoC phases in the SiO_2_-supported catalyst, could result in interphases regions with appropriate characteristics to enhance RWGS on Mo_x_C/SiO_2_ catalyst. In this context, for different Mo_x_C bulk catalysts, the lowest activation energy in the RWGS was found for a catalyst containing several Mo_2_C and MoC phases [31].

All the supported catalysts showed high CO selectivity values. When CO_2_:H_2_ = 1/3 was used, CO selectivity were always higher than 92% (Figure 8B). The highest CO selectivity was observed for the Mo_x_C/SiO_2_ catalyst, achieving at 673 K, 98.5%. Only Mo_x_C/Al_2_O_3_ showed CO selectivity values slightly lower than that of unsupported Mo_2_C (Figure 8B). CH_4_ was the main byproduct and only very small amounts of ethylene were formed. 

For a proper comparison of the catalysts, the values of CO production were calculated per mol of Mo in the samples; results are shown in Figure 9. All the supported catalysts showed a higher CO production per mol of Mo compared to the unsupported Mo_2_C catalyst [21]. At the end of the catalytic test, Mo_x_C/SiO_2_ and Mo_x_C/Al_2_O_3_ showed a higher CO production at 648 K than before reaction at 673 K (Figure 9). This could be related with the removal of remaining oxygen surface species during the reaction at 673 K. The highest CO production in the whole range of reaction temperature tested was obtained for Mo_x_C/SiO_2_; it reached about 17.0 mol CO/mol Mo·h at 673 K. 

**Figure 10:**
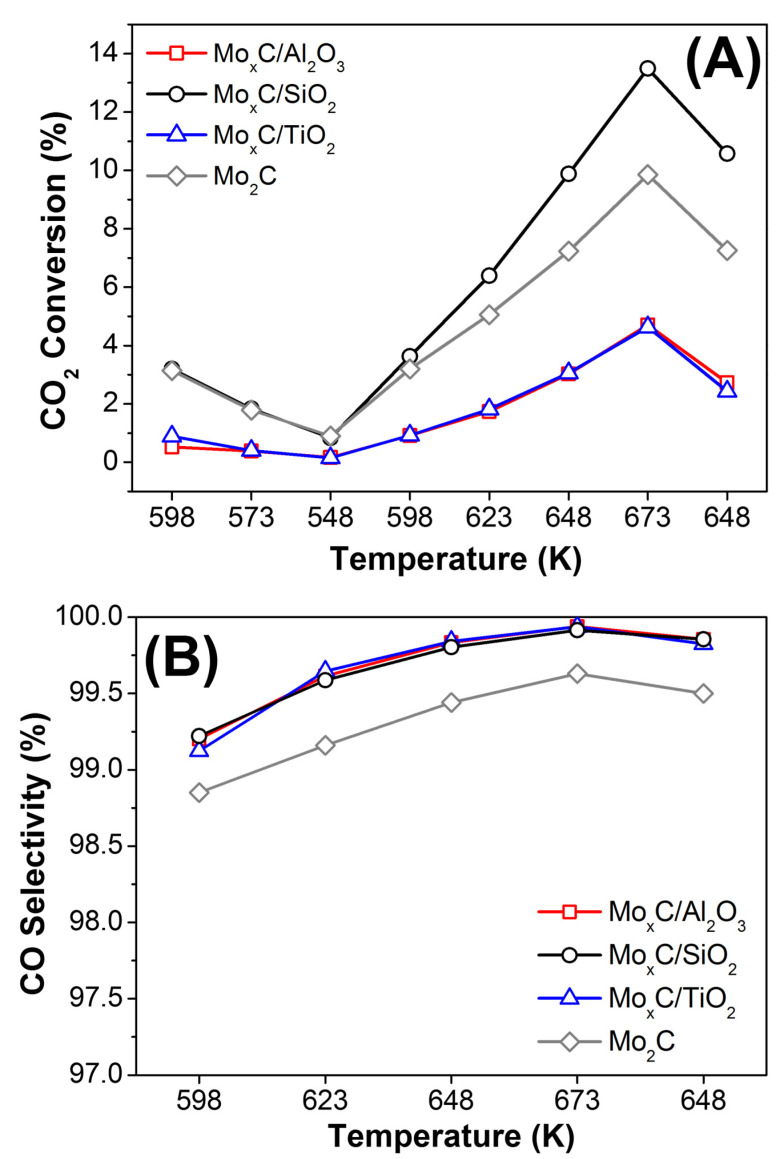
Catalytic behavior of Mo_x_C/support and unsupported reference Mo_2_C catalysts in the RWGS reaction as a function of reaction temperature; (**A**) CO_2_ conversion, (**B**) CO selectivity. Reaction conditions: m_cat_ = 150 mg, CO_2_/H_2_/N_2_ = 1/1/3, GHSV = 3000 h^−1^, P = 0.1 MPa.

**Figure 11:**
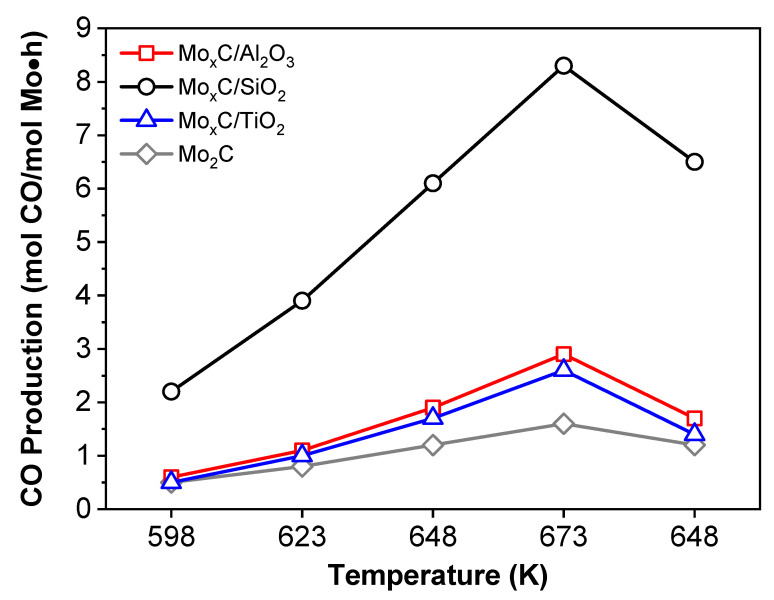
CO production per mol Mo as a function of reaction temperature in RWGS over Mo_x_C/support and unsupported reference Mo_2_C catalysts. Reaction conditions: m_cat_ = 150 mg, CO_2_/H_2_/N_2_ = 1/1/3, GHSV = 3000 h^−1^, P = 0.1 MPa.

Catalysts were also tested in the RWGS using a stoichiometric ratio of the reactant mixture, CO_2_/H_2_/ = 1/1. Figure 10 shows the variation of CO_2_ conversion and CO selectivity values. As expected, the CO_2_ conversion (Figure 10A) was lower and the CO selectivity (Figure 10B) higher when a mixture CO_2_/H_2_ = 1/1 was used than when the reactant mixture was CO_2_/H_2_ = 1/3. Using the CO_2_/H_2_ = 1/1 reactant mixture, the highest CO_2_ conversion (Figure 10A) and the highest CO production per mol of Mo (Figure 11), in the whole range of reaction temperature tested, were also found over the Mo_x_C/SiO_2_ catalyst. In this case, at the end of the catalytic test, only for Mo_x_C/SiO_2_ a slightly higher CO production at 648 K than before reaction at 673 K was observed (Figure 11).

It is noteworthy, that after the overall RWGS study carried out, all supported catalysts, showed quite constant values of CO_2_ conversion and CO selectivity during the last step at 648 K (5 h), under both CO_2_/H_2_/ = 1/3 and CO_2_/H_2_/ = 1/1 conditions.

The apparent activation energies (E_a_) for CO production over supported catalysts were calculated according to the Arrhenius plots in the temperature range of 598–648 K; values between 65–78 kJ/mol were obtained (Table 2). These values are in the range of that recently reported for an alumina supported Mo_2_C cluster-based catalyst (76.4 kJ/mol) [39]. Mo_x_C/SiO_2_ showed the lowest E_a_ for CO production. As stated above, the best performance of Mo_x_C/SiO_2_ could be related with the coexistence in this catalyst of different Mo_x_C phases, hexagonal Mo_2_C and cubic MoC, as has been recently suggested for unsupported Mo_x_C catalysts [31]. Moreover, Mo_x_C/SiO_2_ showed the highest contribution of Mo^2+^/Mo^3+^ and Mo^4+^ species to the total surface Mo^n+^ species. For Mo_x_C-based catalysts, an easy reduction under reaction conditions of molybdenum species has been related with their performance in RWGS [41].

Post-reaction catalysts were characterized by BET and XRD. Only a slight decrease in the BET surface area was found after the RWGS reaction (Table 1). The XRD patterns of fresh (Figure 1, Figure 2 and Figure 3) and post-reaction catalysts after the test with CO_2_/H_2_ = 1/3 (Figure S3) were similar. Meanwhile, the presence of MoO_2_ was detected by XRD in post-reaction Mo_x_C/SiO_2_ and Mo_x_C/TiO_2_ when the reactant mixture was CO_2_/H_2_ = 1/1 (Figure S4); the oxidation could be prevented under a richer hydrogen atmosphere (CO_2_/H_2_ = 1/3) due to an easier removal of the O surface species formed from the CO_2_ activation over these materials under CO_2_/H_2_ = 1/3 conditions [21,31].

## 4. Conclusions

Using urea and MoCl_5_ as carbon and molybdenum sources, different Mo_x_C phases were successfully supported over Al_2_O_3_, SiO_2_ and TiO_2_. The support determined the developed Mo_x_C phases on the materials and their catalytic behavior in the RWGS. Hexagonal Mo_2_C nanoparticles on Mo_x_C/Al_2_O_3_ and cubic MoC nanoparticles on Mo_x_C/TiO_2_ were found. Over Mo_x_C/SiO_2_ both hexagonal Mo_2_C and cubic MoC nanoparticles were present. In all cases, supported hexagonal Mo_2_C nanoparticles were larger than cubic MoC ones. 

All catalysts showed a stable catalytic behavior and exhibited higher CO production per mol of Mo than the unsupported hexagonal Mo_2_C similarly prepared, under the reaction conditions used (CO_2_/H_2_ = 1/3 and CO_2_/H_2_ = 1/1; T = 548–673 K).

Mo_x_C/SiO_2_ exhibited the highest surface ratio of Mo species with low oxidation states (Mo^2+,3+,4+^) and the best performance in the RWGS reaction. Over Mo_x_C/SiO_2_, CO_2_ conversion of 27.5% and CO selectivity of 98.5% were achieved at 673 K under CO_2_/H_2_ = 1/3; for CO production, an apparent activation energy of 64.9 ± 3.2 kJ mol^−1^ was determined at 598–648 K under CO_2_/H_2_ = 1/1. The catalytic behavior is proposed to be governed by the supported Mo_x_C phase. The simultaneous presence of hexagonal Mo_2_C and cubic MoC nanoparticles in Mo_x_C/SiO_2_ plays a main role on the catalytic behavior of this catalyst.

## Figures and Tables

**Table 1:** Mo content, determined by chemical analysis and surface area (S_BET_) of fresh and post-reaction catalysts.

Catalyst	Mo (%wt)	S_BET_ (m^2^ g^−1^)		
		Fresh ^a^	Post-Reaction ^b^	Post-Reaction ^c^
Mo_x_C/Al_2_O_3_	25.1	119 (204)	93	97
Mo_x_C/SiO_2_	25.5	129 (181)	115	107
Mo_x_C/TiO_2_	27.5	39 (13)	32	25

^a^ between brackets S_BET_ of supports treated at 1073 K; ^b^ CO_2_/H_2_/N_2_ = 1/3/1 reactant mixture; ^c^ CO_2_/H_2_/N_2_ = 1/1/3 reactant mixture.

**Table 2:** Apparent E_a_ determined for Mo_x_C/support catalysts and surface characteristics determined from XPS.

Catalyst	E_a_ (kJ·mol^−1^)	(Mo^2+,3+^/Total Mo^n+^)_XPS_	(Mo^2+,3+,4+^/Total Mo^n+^)_XPS_
Mo_x_C/Al_2_O_3_	77.7 ± 1.7	0.277	0.347
Mo_x_C/SiO_2_	64.9 ± 3.2	0.431	0.690
Mo_x_C/TiO_2_	77.9 ± 4.1	0.098	0.316

Reaction conditions: CO_2_/H_2_/N_2_ = 1/1/3, GHSV = 3000 h^−1^, P = 0.1 MPa and T = 598–648 K.

## Data Availability

The data present in this study are available on request from the corresponding author.

## Supplementary Materials

The following supporting information can be downloaded at: https://www.mdpi.com/article/10.3390/nano12183165/s1, Figure S1. Raman spectra of fresh Mo_x_C/support catalysts; Figure S2. XP spectra of Mo_x_C/support catalysts. (A) Al 2p level registered for Mo_x_C/Al_2_O_3_, (B) Si 2p level registered for Mo_x_C/SiO_2_, (C) Ti 2p level registered for Mo_x_C/TiO_2_; Figure S3. XRD patterns of Mo_x_C/support catalysts after RWGS reaction (CO_2_/H_2_ = 1/3); reaction conditions: m_cat_ = 150 mg, GHSV = 3000 h^−1^, P = 0.1 MPa.; Figure S4. XRD patterns of Mo_x_C/support catalysts after RWGS reaction (CO_2_/H_2_ = 1/1); reaction conditions: m_cat_ = 150 mg, GHSV = 3000 h^−1^, P = 0.1 MPa.
