# Supplementary material for: Supported Nanostructured MoxC Materials for the Catalytic Reduction of CO2 through the Reverse Water Gas Shift Reaction
Source: Nanomaterials (Basel). 2022 Sep 13;12(18):3165. doi: 10.3390/nano12183165 (PMC9506042; doi:10.3390/nano12183165)
Supplement: Supplementary file 1 [file nanomaterials-12-03165-s001.zip › nanomaterials-1861928-supplementary.pdf]

SUPPLEMENTARY INFORMATION

Supported Nanostructured Mo_xC Materials for the Catalytic Reduction of CO_2 through the Reverse Water Gas Shift Reaction

Arturo Pajares [1,2,†], Xianyun Liu [1,‡], Joan R. Busacker [1], Pilar Ramírez de la Piscina [1] and Narcís Homs [1,2,*]

[1] Departament de Química Inorgànica i Orgànica, Secció de Química Inorgànica & Institut de Nanociència i Nanotecnologia (IN2UB), Universitat de Barcelona, Martí i Franquès 1, 08028 Barcelona, Spain

[2] Catalonia Institute for Energy Research (IREC), Jardins de les Dones de Negre 1, 08930 Barcelona, Spain

* Correspondence: nhoms@irec.cat or narcis.homs@qi.ub.edu

† Present address: Sustainable Materials Management, Flemish Institute for Technological Research (VITO NV), Boeretang 200, 2400 Mol, Belgium.

‡ Present address: College of Chemistry and Chemical Engineering, Henan Polytechnic University, Jiaozuo 454003, China.

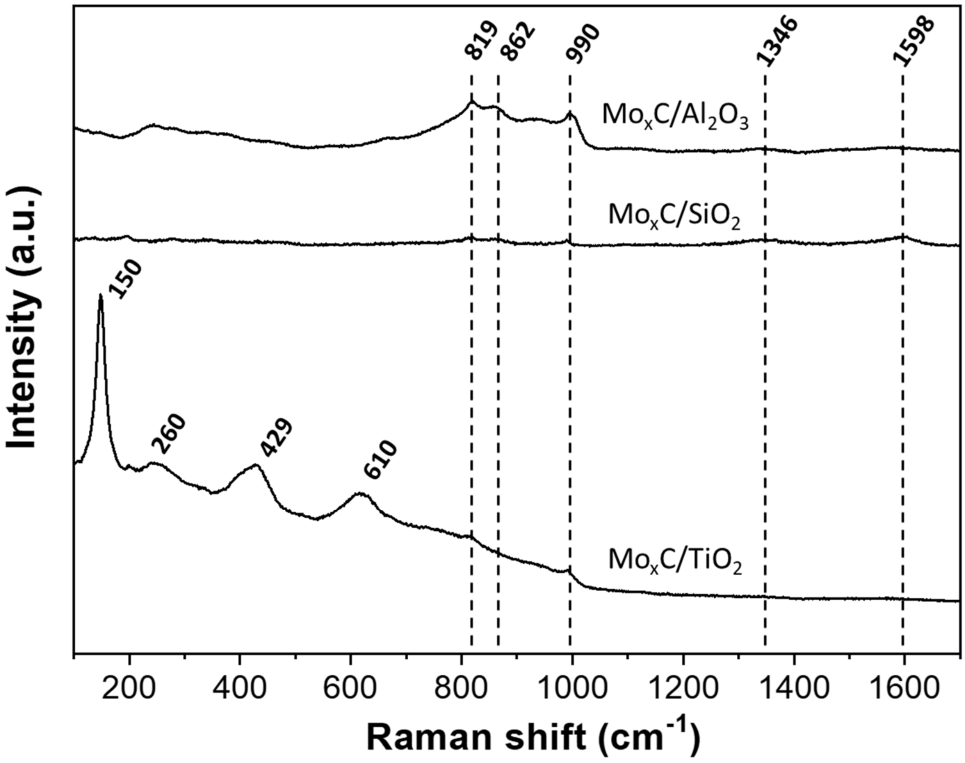

Figure S1. Raman spectra of fresh Mo_xC/support catalysts.

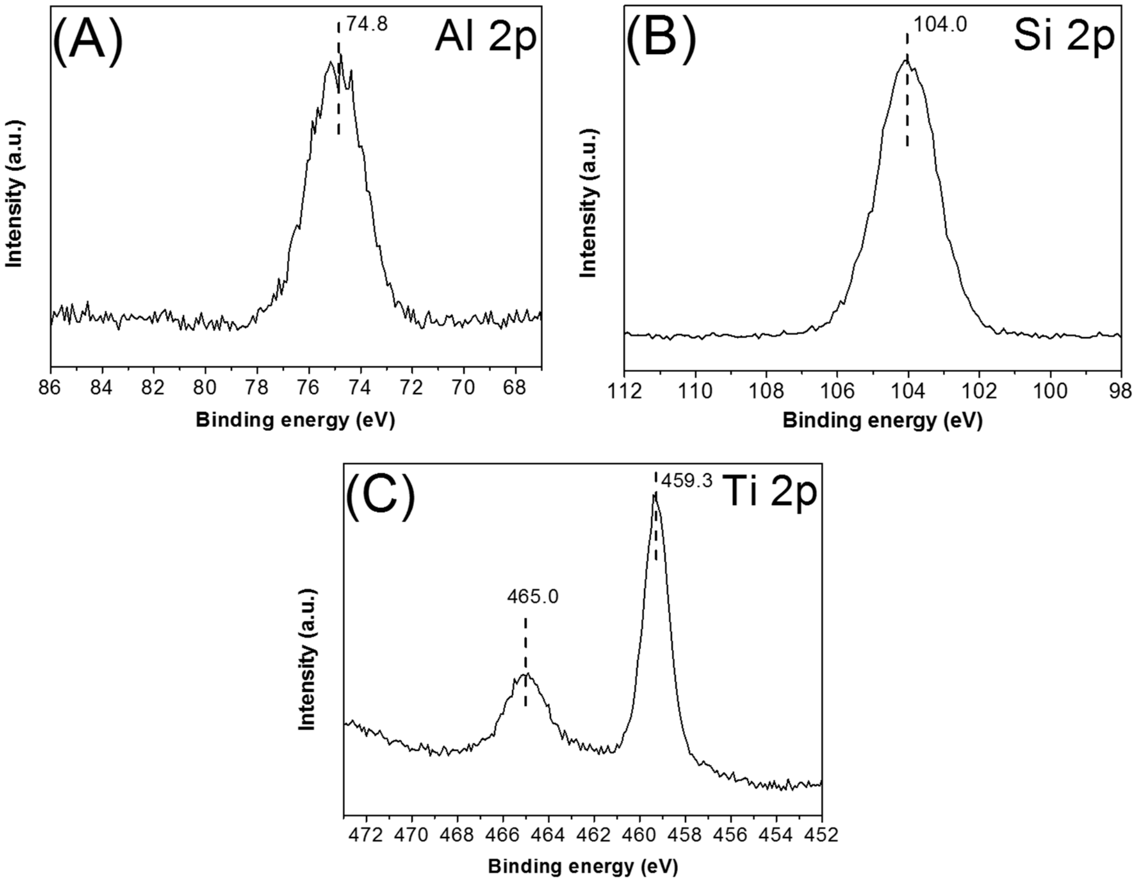

Figure S2. XP spectra of Mo_xC/support catalysts. (A) Al 2p level registered for Mo_xC/Al_2O_3, (B) Si 2p level registered for Mo_xC/SiO_2, (C) Ti 2p level registered for Mo_xC/TiO_2.

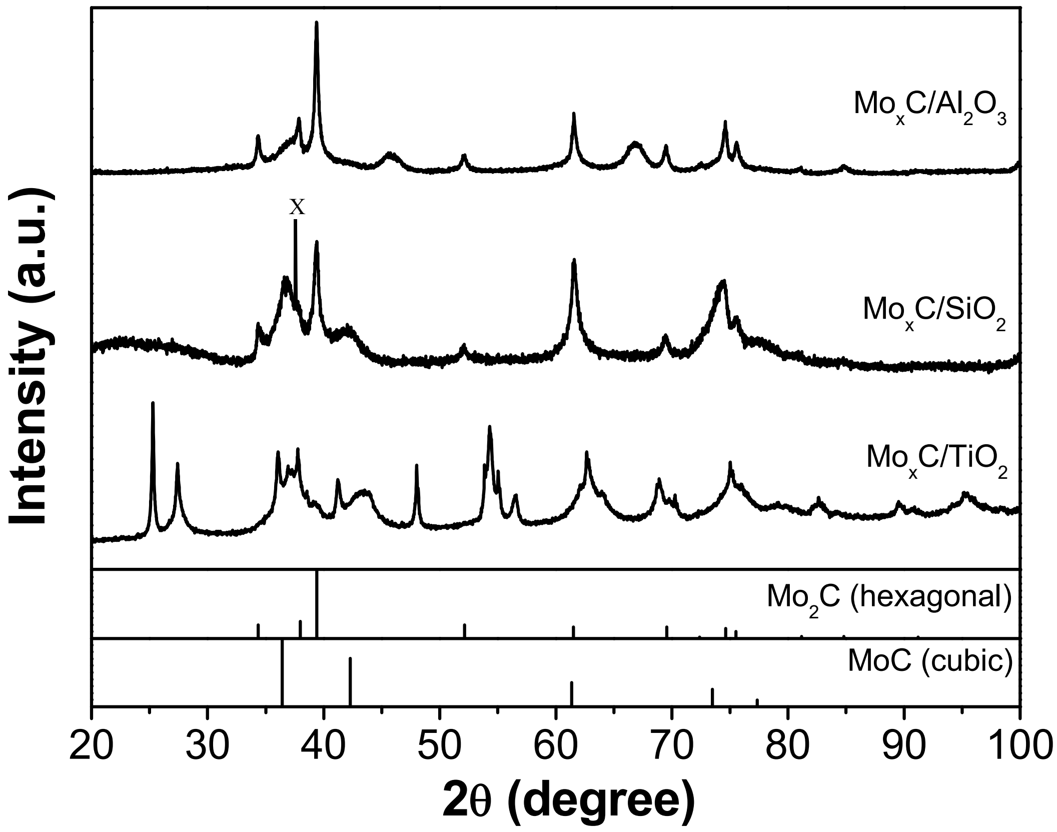

Figure S3. XRD patterns of Mo_xC/support catalysts after RWGS reaction (CO_2/H_2=1/3); reaction conditions: m_{cat}=150 mg, GHSV=3000 h^{-1}, P=0.1 MPa. X: signal due to SiC from catalytic bed.

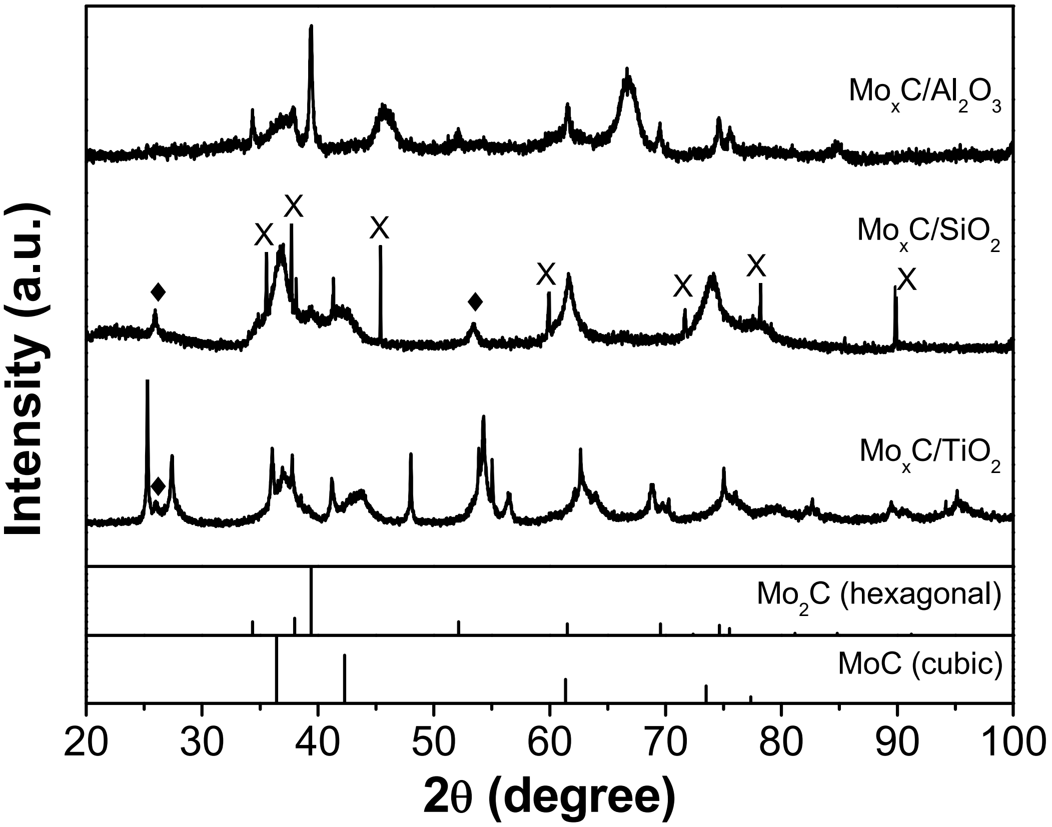

Figure S4. XRD patterns of Mo_xC/support catalysts after RWGS reaction (CO_2/H_2=1/1); reaction conditions: m_{cat}=150 mg, GHSV=3000 h^{-1}, P=0.1 MPa. ♦: MoO_2; X: signal due to SiC from catalytic bed.
